# Reminder system for health screening in early childhood – an analysis regarding different social circumstances

**DOI:** 10.1186/s12887-021-02917-4

**Published:** 2021-10-07

**Authors:** Simone Weyers, Annika Höhmann, Simon Götz, Katharina Kreffter

**Affiliations:** Institut für Medizinische Soziologie, Centre for Health and Society, Medizinische Fakultät, Heinrich-Heine-Universität Düsseldorf, Düsseldorf, Germany

**Keywords:** Child health, Social inequalities, Prevention

## Abstract

**Background:**

Children with a low socio-economic position (SEP) participate in prevention and health examinations less often. In order to increase participation, reminder systems have been implemented in Germany since 2009. The aim of the study is to investigate whether this implementation is associated with an increased participation in health examination in early childhood for children in disadvantaged social circumstances.

**Methods:**

We used data from the school enrolment examination from 2002 to 2017 from the city of Duesseldorf (*n* = 64,883 children). With a trend analysis we observed health examination over time and we compared rates of children after implementation of the reminder system (2010 or later) to those who were not exposed to the programme (earlier than 2010). Health examination was measured by participation in the last examination before school entry (“U9”) documented by paediatricians. Social circumstances included neighbourhood deprivation (very high to very low), migration background (foreign first language vs. German) and family status (one-parent vs. two-parent families). Poisson regression estimated adjusted Prevalence Ratios (PR) with a 95% confidence interval (CI) of U9 participation by reminder system exposure, both for the total population and within groups of social circumstances. Based on that, we calculated adjusted participation rates (predictive margins) by reminder system exposure for the different social circumstances.

**Results:**

Participation rates increased slightly, but gradually over time. The probability of U9 participation for children exposed to the reminder system is 1.04-fold (1.03–1.04 CI) compared to children who were not exposed to it. The association of the reminder system and U9 participation differs according to social circumstances. Adjusted prevalences increased the most in the group of children from very deprived neighbourhoods, ranging from 84.3 to 91.4% (PR = 1.07; 1.03–1.10 CI); in all language groups; more in children from one-parent families ranging from 82.4 to 88.9% (PR = 1.07; 1.05–1.09 CI).

**Conclusion:**

Our results suggest that reminder systems have a moderate impact on the participation in health examinations in early childhood in the general population. In vulnerable groups, however, they could make a difference. Reminder systems should be combined with further activities of tailored prevention.

## Background

Children with a low socio-economic position (SEP) have a higher risk of poor general health and health constraints than their peers with a higher SEP [1, 2]. At the same time they participate less often in prevention and health promotion, among which are the nine health examinations “U1 – U9” in early childhood that are provided free of charge in Germany by outpatient paediatricians [3–5].

These “U-examinations” aim to identify health problems in a timely manner and to initiate health promoting measures for the child. To this end, nine examinations are carried out in a given time window from U1 at birth to U9 in the fifth year of live. Each examination has a have specific focus, e.g. physical development, language development or vaccination status. Another purpose of the U-examination is to detect situations of social emergency and child welfare risk. Against the background of several cases of child abuse in Germany in 2006 and 2007, a Federal law was created in order to increase participation in U-examinations. Most German States implemented a mandatory invitation, reminding and reporting system [6]. If a family has not recuperated the missed U-examinations upon further request, a central organisation transfers the case to the local health or youth welfare authorities. These approach the family, where necessary by means of a home visit, inform them about the aim and procedure of the U-examination and offer a subsidiary examination by a public medical officer [6]. In the German State of North Rhine-Westphalia, a reminder system was implemented in 2010, where participation in U5 to U9 was checked and parents were reminded to participate if necessary [7].

Reminder systems for child health promotion have been predominantly used in terms of health screening and immunisation. According to the review of Jacobson Vann et al. [8] including 75 studies reminding people, e.g. by calls, cards or test messages, increases participation in vaccinations, also in children. First experiences in the German Federal States also showed that, after implementation of the Federal law, participation increased, especially from 4 years of age onwards [9]. Also, an evaluation in North Rhine-Westphalia based on administrative data showed that participation in U5 to U9 generally increased [7]. Given the above-mentioned inequalities in child health, it is important to examine if also children with a low socio-economic position benefit from reminder systems. In the North Rhine-Westphalian study a social comparison was made on a district group level with the result that there is an association of unemployment and participation rates in the U-examinations (Ibidem, p. 4). Furthermore, in a survey of paediatricians in the State of Schleswig-Holstein, an increased participation was observed in socially disadvantaged families and those with a migration background [10]. However, social differential analyses of reminder systems in child health based on individual and objective data have been lacking so far. The aim of the study was therefore to investigate if the implementation of a reminder system is associated with an increased participation in health examination in early childhood for children in disadvantaged social circumstances. We did so using administrative data of the city of Duesseldorf.

## Methods

We used anonymised data of the school enrolment examination (SEE) that is mandatory for each child before school entry. It is carried out by public medical officers, paediatricians working in the public health service of the community. The primary aim of this examination is to detect health and developmental disorders that are relevant for school success and to advise parents regarding therapy [11]. Moreover, vaccination status and participation in health examinations and therapies are assessed. The SEE includes families from all social circumstances and, therefore, allows social differential analyses of different aspects of child health [12, 13]. The present study comprises full samples of 16 SEE cohorts from 2002 to 2017 from the city of Duesseldorf with a total of 73,457 children. With a trend analysis with repeated cross sectional studies we observed health examination over time and we compared health examination rates of children after implementations of the reminder system in North Rhine-Westphalia to those that were not yet exposed to the programme.

Variables were measured as follows: *Health examination* was operationalised using the example of the U9, the last examination before school entry. U9 participation was taken from the child’s “yellow booklet” where paediatricians document all U-examinations. The *reminder system programme* was approximated by the year 2010 where implementation was fully completed [7]. Since the U9 examination and school enrolment examination are both conducted at 6 years of age, children with SEE in 2010 or later were defined as exposed. Due to data protection, the administrative data does not include regular indicators of a child’s socio-economic position such as a parental education, occupation and income. However, three indicators for disadvantaged social circumstances in the context of child health [14–16] were available: (i) *neighbourhood deprivation* was defined by the socio-spatial degree of deprivation for children’s residential addresses. Based on indicators such as unemployment and living space per person the local authorities classified 166 social spaces into five neighbourhood types ranging from very high to very low deprivation [17]. This construction of such types is meanwhile common in larger German cities [18] and it recognizes evidence-based markers of economic disadvantage [19]; (ii) *migration background* was operationalised by the child’s first language that was assessed by public medical officers in the SEE anamnestic interview. We compared Yugoslav, Turkish, Russian, Moroccan and other first languages to German; (iii) growing up in *one-parent-families.* Family status was also assessed in the SEE interview and we compared one-parent to two-parent families.

All children who presented the yellow booklet with information on U-examinations were included in the analyses. First, we calculated U9 participation rates for each cohort and according to social circumstances. Then, we used Poisson regression [20] to estimate Prevalence Ratios (PR) with a 95% confidence interval (CI) of U9 participation by reminder system exposure, both for the total population and within groups of social circumstances (adjusted for age, sex and the other indicators of social circumstances). Based on that we calculated adjusted participation rates (predictive margins [21];) by reminder system exposure for the different social circumstances. Finally, in order to test differences in the increase of participation rates between social groups we calculated multiplicative interaction terms and performed a Wald test (not shown). All analyses were conducted using Stata 14.

## Results

Sixty-four thousand eight hundred eighty-three children were included in the analyses. Overall, 89.3% of children participated in the U9 examination and 43.4% were exposed to the reminder system. With 27.0%, more than one fourth of children lived in a (very) deprived neighbourhood, 35.1% did not speak German as their first language and 12.5% grew up in a one-parent family (Table 1). The large number of missing values in the latter two variables results from the fact that both were not available for three cohorts (see also Table 2).

**Table 1:** Sample characteristics

Variable (missing values)	Categories	n (mean)	% (SD)
Age (366)		(5.58)	(0.50)
Sex (1)	Male	33,323	51.4
	Female	31,559	48.6
U9 participation (0)	No	6978	10.8
	Yes	57,905	89.3
Reminder system (0)	Not exposed	36,712	56.6
	Exposed	28,171	43.4
Neighbourhood deprivation (1.995)	Very low	5531	8.5
	Low	16,408	25.3
	Middle	23,474	36.2
	High	13,808	21.3
	Very high	3667	5.7
First language (13.916)	German	31,180	48.1
	Other	10,869	21.3
	Yugoslav	1392	2.2
	Turkish	3569	5.5
	Russian	1962	3.0
	Moroccan	1995	3.1
Family status (12.073)	Two-parent family	44,721	68.9
	One-parent family	8089	12.5
**Total**		**64,883**	**100.00**

*N* Number, *SD* Standard deviation

**Table 2:** U9 participation rates (%) by social circumstances

Year	2002	2003	2004	2005	2006	2007	2008	2009	2010	2011	2012	2013	2014	2015	2016	2017	Total	perc.diff.^a^
**Neighbourhood deprivation**																		
Very low	93.0	91.6	87.6	89.8	91.6	90.0	91.0	88.0	87.0	92.5	92.0	94.4	91.0	92.2	96.4	95.1	**91.8**	2.1
Low	90.6	92.2	91.9	90.8	90.4	90.7	92.2	89.7	90.2	91.4	92.6	93.4	94.7	94.4	94.7	94.6	**92.1**	4.0
Middle	87.2	87.8	87.6	87.1	88.8	90.4	89.3	87.8	88.7	91.4	90.5	91.1	93.0	92.4	91.6	92.8	**89.8**	5.6
High	80.9	83.8	83.7	85.1	84.0	86.5	86.3	84.2	85.5	84.9	83.8	84.7	88.8	85.5	89.0	90.1	**85.4**	9.2
Very high	67.5	79.0	79.7	81.7	77.8	82.3	86.1	82.0	90.5	90.4	87.1	84.5	89.5	86.1	90.2	88.9	**84.4**	21.4
**First language**																		
German	m.v.	m.v.	89.5	89.8	89.2	91.2	90.2	89.1	90.5	92.3	91.1	m.v.	93.8	93.4	94.3	95.0	**91.4**	5.5
Other	m.v.	m.v.	87.3	84.3	86.7	85.6	85.9	84.2	85.4	85.4	87.5	m.v.	89.6	87.8	88.6	89.9	**87.0**	2.6
Yugoslav	m.v.	m.v.	72.6	74.5	80.0	83.8	85.8	85.3	86.8	84.6	85.0	m.v.	84.6	90.8	91.1	86.4	**83.6**	13.8
Turkish	m.v.	m.v.	81.4	85.5	84.9	86.6	89.0	84.3	86.4	89.8	87.2	m.v.	89.7	89.7	94.4	89.1	**87.1**	7.7
Russian	m.v.	m.v.	78.3	87.8	84.6	87.4	86.4	86.5	85.2	88.1	90.6	m.v.	92.9	93.5	91.5	91.5	**88.4**	13.2
Moroccan	m.v.	m.v.	76.7	81.5	85.7	81.5	90.6	81.0	83.3	86.5	86.0	m.v.	90.4	85.6	86.8	91.8	**85.2**	15.1
**Family status**																		
Two-parent family	87.2	88.2	88.3	89.3	89.3	89.9	90.1	88.2	89.0	90.7	m.v.	91.3	m.v.	m.v.	92.6	92.9	**89.7**	5.7
One-parent family	80.1	81.2	80.7	79.7	80.4	86.0	84.5	81.8	85.8	85.8	m.v.	86.0	m.v.	m.v.	88.8	92.4	**84.3**	12.3
**Total**	**86.2**	**87.9**	**87.3**	**87.7**	**87.8**	**89.2**	**89.1**	**87.1**	**88.4**	**89.9**	**89.7**	**90.4**	**92.0**	**91.1**	**92.0**	**92.8**	**89.3**	6.6

^a^ percentage difference; *m.v*. Missing value

**Fig. 1:**
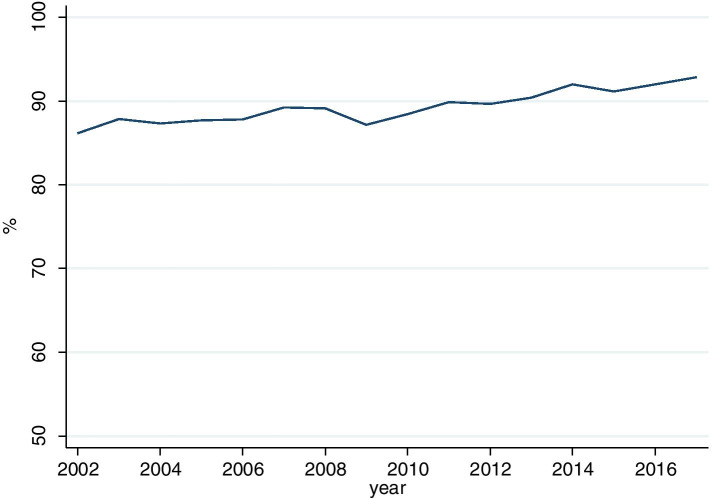
Participation rates in the total population

Figure 1 shows the U9 participation rates by cohorts. With rates increasing from 86.2% in 2002 to 92.8% in 2017 we observed a slight upward trend by 7% percentage points.

Table 2 displays U9 participation rates according to social circumstances. The percentage difference from 2002 to 2017 within groups of neighbourhood deprivation increases from 2.1% in the least deprived group to 21.4% in the most deprived group. Also, an increase in U9 participation is observed not only in children with German as their first language but also, and quite steeper, in children with a foreign first language. Finally, with 12.3% in one-parent families, the increase is higher when compared with two-parent families with 5.7%.

For further analyses the cohorts were grouped into those children exposed to the implemented programme and those who were not. Table 3 shows that the probability of U9 participation for children exposed to the reminder system is 1.04-fold (1.03–1.05 CI) compared to children who were not exposed. This association is adjusted for age, sex and social circumstances. Moreover, the latter are associated with U9 participation: With an PR = 0.97 (0.95–0.99 CI) children from very deprived neighbourhoods have a lower probability for U9 participation compared to those from well-off neighbourhoods; children with a foreign first language have a lower probability than their peers with German as their first language; children who have grown up in one-parent families than their peers from two-parent families (PR = 0.94; 0.93–0.95 CI).

**Table 3:** Prevalence Ratios (PR) with a 95% Confidence Interval (CI) for U9 participation

U9 participation	PR	95% CI
No reminder system	Reference	
Reminder system	**1.04**	**1.03–1.05**
Neighbourhood deprivation: very low	Reference	
Low	1.00	1.00–1.02
Medium	1.00	0.99–1.01
High	**.97**	**0.96–0.98**
Very high	**.97**	**0.95–0.99**
First language: German	Reference	
Other	**.95**	**0.94–0.96**
Yugoslav	**.92**	**0.90–0.95**
Turkish	**.96**	**0.95–0.98**
Russian	**.96**	**0.94–0.98**
Moroccan	**.93**	**0.91–0.95**
Family status: Two-parent family	Reference	
One-parent family	**.94**	**0.93–0.95**

Significant results in bold letters

**Fig. 2:**
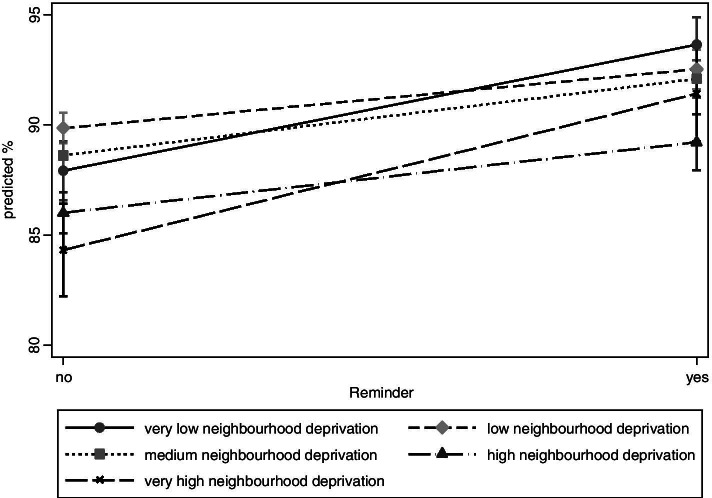
Adjusted prevalences of U9 participation by neighbourhood deprivation

The following three figures recapitulate that the association of a reminder system and U9 participation differs according to social circumstances. Figure 2 shows that, after programme implementation, the adjusted prevalences of U9 participation increase the most in the group of children from very deprived neighbourhoods, ranging from 84.3 to 91.4% (PR = 1.08; 1.05–1.12 CI; results not shown). In the group of children from deprived neighbourhoods the increase from 86.0 to 89.2% is less steep (PR =1.03; 1.12–1.05 CI). A strong increase from 87.9 to 93.7% is also observed in the group of children from the least deprived neighbourhoods (PR = 1.07; 1.05–1.09 CI). However, group differences are statistically not significant. Results are adjusted for age, sex and the other indicators of social circumstances.

**Fig. 3:**
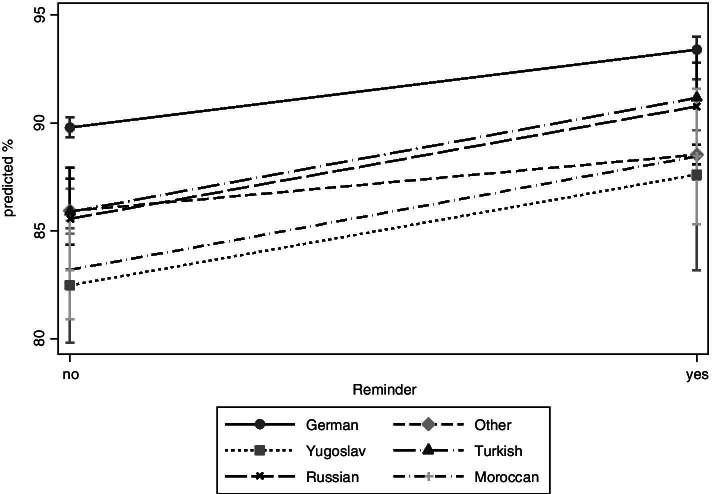
Adjusted prevalences of U9 participation by migration background (first language)

Stratified by first language, an increase of U9 participation is observed in all language groups (Fig. 3). It ranges from 89.8 93.4% in children with German as their first language (PR = 1.04; 1.03–1.05 CI; results not shown); from 82.5 to 87.6% (PR = 1.07; 1.01–1.14 CI) in children with Yugoslav as their first language; from 85.9 to 91.2% (PR = 1.06; 1.03–1.09 CI) in children with Turkish as their first language; from 85.6 to 90.8% (PR = 1.06; 1.02–1.10 CI) in children with Russian as their first language and from 83.2 to 88.5% (PR = 1.07; 1.02–1.11 CI) in children with Moroccan as their first language. Again, group differences are statistically not significant.

**Fig. 4:**
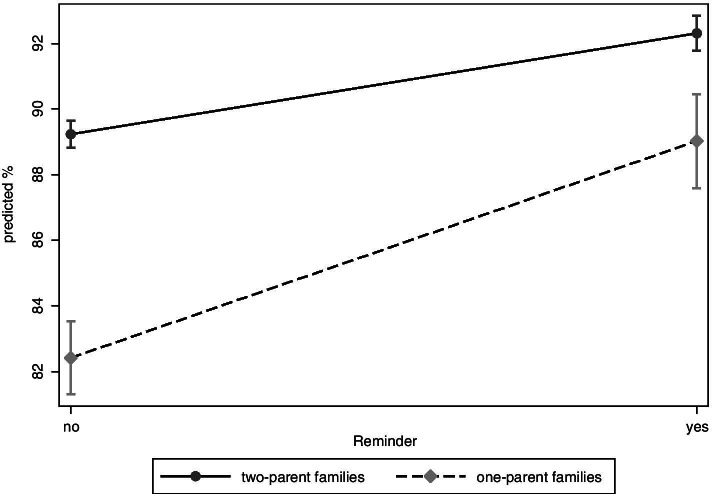
Adjusted prevalences of U9 participation by family status

Finally, the increase of U9 participation differs by family status (Fig. 4). While adjusted prevalences increase from 89.2 to 92.3% (PR = 1.04; 1.03–1.04 CI) in children who have grown up in two-parent families, it increases from 82.4 to 89.0% (PR = 1.08; 1.06–1.10 CI) in children from one-parent families. In this case, group differences are statistically significant.

## Discussion

The aim of the study was to investigate if the implementation of a reminder system is associated with an increased participation in health examination in early childhood for children in disadvantaged social circumstances. Overall, participation rates have increased slightly, but gradually over time. The increase of 86.2% in 2002 to 92.8% in 2017 is less than that reported in other studies. In the German Health Interview and Examination Survey for Children and Adolescents (“KiGGS”) full participation in the U-examinations has increased from 81.6% in the baseline study (2003–2006) [22] to 97.2% in wave 2 (2014–2017) [23]. Also, we did not observe a distinct increase after 2010 in our data as we would have expected after full implementation of the reminder system. It is argued that administrative procedures are optimised step by step [7]. Another explanation for the gradual increase even before programme implementation could be the range of existing community activities: Health insurance companies and authorities are important players in the community system, which transfer families to existing prevention programmes. One health insurance company reported upon request that they had been sending out information brochures since 1996 and personalised letters since 2006. The youth welfare authorities have been visiting all parents of a first born child since 2009 in order to inform them about child development and prevention programmes [24].

In the frame of the social-differential analysis we found several effects: First, the steepest increase was observed in children from very deprived neighbourhoods. This could be explained by the fact that the baseline value was especially low in this group. Also, the reminder system was accompanied by an intensified prevention in Duesseldorf neighbourhoods with special needs since 2012. In the frame of a North Rhine-Westphalian State programme (“No child left behind” [25]) local prevention managers had been implemented in neighbourhoods with special needs. These managers were in close contact with families and transferred them to existing prevention offers. Second, an increase of participation rates was observed for all language groups. It is widely acknowledged that language barriers, unfamiliarity with the health care system and gaps in health literacy impair the accessibility of health services for migrants [26]. Third, a strong increase in participation was observed for children who grow up in one-parent families. There is some evidence that single parents have an increased risk of non-participation in preventive child health examinations [27, 28] and incomplete immunisation schedules [29, 30]. One hypothesis is the time limit of single parents [28, 30] and the increase after implementation of the reminder system could be explained by the fact that the examination was simply forgotten.

### Limitations

A central limitation is the operationalisation of the reminder system by implementation year. The reminder was only sent in case of a missed U-examination. If and how families were actually reached could not be assessed by individual data. Furthermore, we could not quantify, which additional factors might have contributed to the increase in participation rates. The concept of the North Rhine-Westphalian reminder system did not foresee direct communication with the target groups, but rather the programme was disseminated by professional associations and communities [7]. Accordingly, additional local measures were necessary that we could not account for.

A strength of the study is that it is based on a large sample. Three different indicators of social circumstances and the U9 participation were objectively measured on an individual level and do not have a substantial bias. This is especially important in the context of a social-differential analysis since the recall bias varies according to sociodemographic factors [4]. Also, due to its naturalistic design, the study’s external validity can be rated as rather high. Replication studies in other German cities should examine whether the association of reminder systems and prevention participation remains under different local circumstances.

## Conclusions

Taken together, our results suggest that reminder systems have a moderate impact on participation in health examination in early childhood. There is some evidence how population groups can be reached by reminder systems: The combination of different communication channels such as a phone call and a letter have proved to be effective in order to promote vaccination in small children [23, 31] and in children with chronic disease [32, 33]. With the spread of mobile phones in hard-to-reach population groups the short message service (SMS) has become important. Thereby, SMS with health relevant information [34] and with interactive components [35] were effective in promoting flu-vaccination in children. In order to recruit urban young populations, a stepwise intervention with a phone call, letter and, if unsuccessful, a home visit was promising [36].

Furthermore, our results give the impression that reminder systems should be combined with further activities of tailored prevention in order to have an impact in socially disadvantaged children. In terms of the three different indicators, the following conclusions can be drawn: With regard to neighbourhood deprivation, it should be noted that many German communities have developed socio-spatial classifications based on data such as living space per inhabitant, nationality of inhabitants or number of households on benefits [18]. With this classification, youth welfare interventions can be prioritised to children who grow up in *very deprived* neighbourhoods. On the other side, children who grow up in (only) *deprived* neighbourhoods, might “fall through the cracks”. With *n* = 13,808 children in deprived neighbourhoods in our sample, this missed chance has concerned a large group and, in the future, further selection criteria should be considered in order to focus on preventive behaviour in vulnerable groups. Regarding migration background with language and knowledge barriers towards the health services, it seems that a reminder system insistently calling attention to a given examination has the potential to increase participation. On the other hand, these language and knowledge barriers could be overcome beforehand. Health systems should provide migrants with information in their language. Also, they should aim to improve the health literacy of migrant families by means of targeted health promotion interventions that take into account the different ways in which people perceive and experience health problems [26, 37]. Accordingly, authoritative and costly recall systems could become redundant to a certain extent.

This is particularly important. Preventive measures adopted by governments might sometimes seem necessary and justified. However, they always present ethical and human rights controversies – even if they are effective [38]. The government is sworn to neutrality with regard to the life styles of its citizens [39]. The German constitutional law foresees that “child care and education are the natural right of parents” (Art. 6 para. 2 GG [40];). Despite the social inequalities in child health and prevention mentioned above, governmental strategies have to be weighed up against cutting these rights. One could argue that in our case the *informational* home visit with the *offer* of a subsidiary health examination is a good compromise.

### Recommendations

Based on our results and the available evidence, we recommend that reminder systems combine different communication channels and that they are complemented with further activities of tailored prevention such as home visits or culturally-sensitive information for as many vulnerable persons as possible.

## Data Availability

The SEE dates analysed during the current study are not publicly available because the data owner is the city of Duesseldorf.

## Abbreviations

SEP: Socio-economic position
SEE: School enrolment examination
PR: Prevalence Ratios
CI: Confidence interval
N: Number
SD: Standard deviation
m.v.: Missing value
